# Supplementary material for: Risk factors for sacrococcygeal pilonidal sinus: a systematic review and meta-analysis supplemented by genetic causal assessment
Source: Front Surg. 2026 Jan 7;12:1718589. doi: 10.3389/fsurg.2025.1718589 (PMC12819706; doi:10.3389/fsurg.2025.1718589)
Supplement: Supplementary file 2 [file Datasheet2.zip › Supplementary Data 2/MR_pipeline_p5e-8/forest_plots/ukb-saige-272.11.finngen_R12_L12_PILONIDALCYST.pdf]

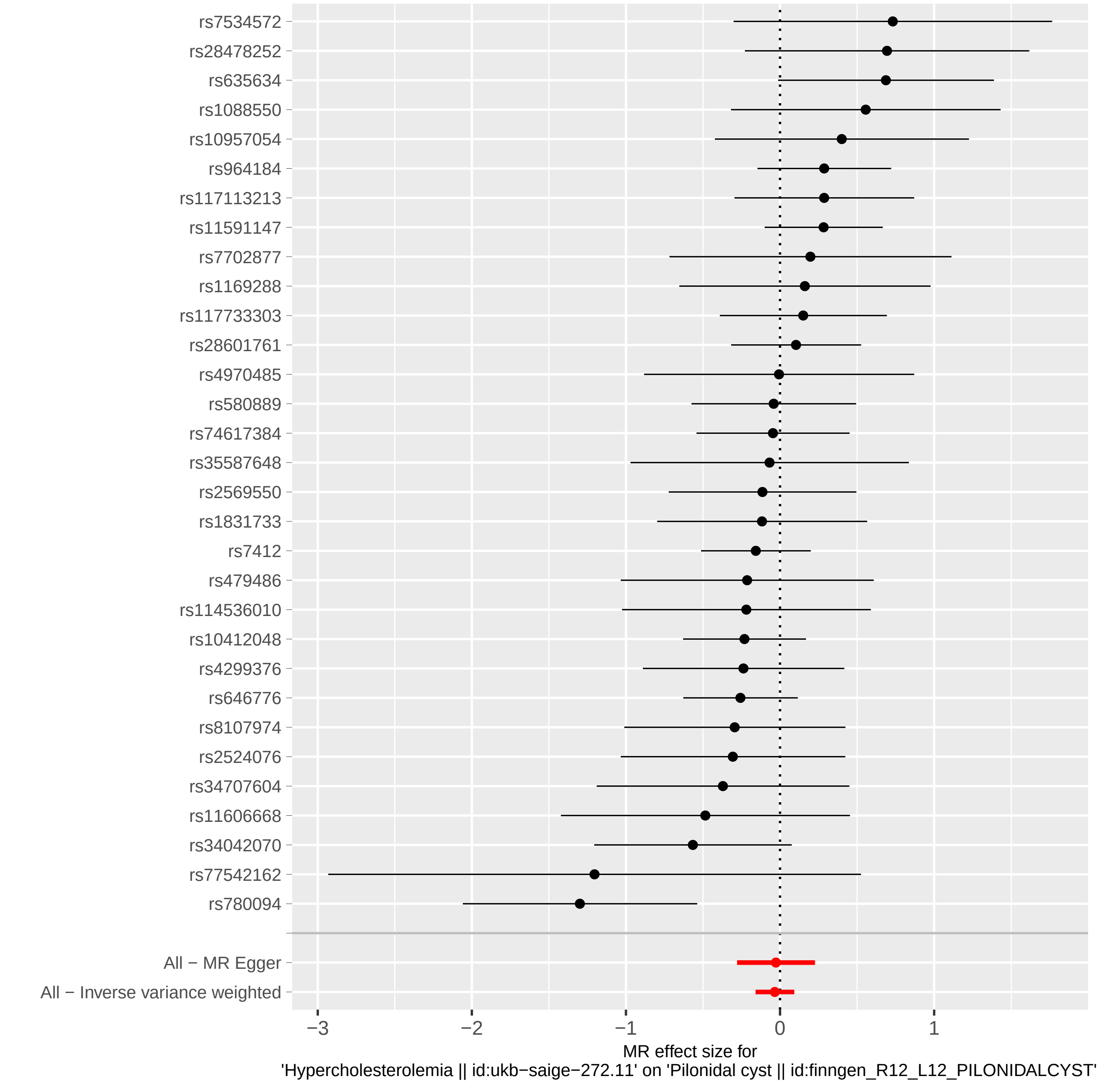
rs7534572
rs28478252
rs635634
rs1088550
rs10957054
rs964184
rs117113213
rs11591147
rs7702877
rs1169288
rs117733303
rs28601761
rs4970485
rs580889
rs74617384
rs35587648
rs2569550
rs1831733
rs7412
rs479486
rs114536010
rs10412048
rs4299376
rs646776
rs8107974
rs2524076
rs34707604
rs11606668
rs34042070
rs77542162
rs780094
All − MR Egger
All − Inverse variance weighted
−3
−2
−1
0
1
MR effect size for
'Hypercholesterolemia || id:ukb−saige−272.11' on 'Pilonidal cyst || id:finngen_R12_L12_PILONIDALCYST'
